# Tau Abnormalities and Autophagic Defects in Neurodegenerative Disorders; A Feed-forward Cycle

**DOI:** 10.31661/gmj.v9i0.1681

**Published:** 2020-01-27

**Authors:** Nastaran Samimi, Akiko Asada, Kanae Ando

**Affiliations:** ^1^Noncommunicable Diseases Research Center, Fasa University of Medical Sciences, Fasa, Iran; ^2^Department of Brain and Cognitive Sciences, Cell Science Research Center, Royan Institute for Stem Cell Biology and Technology, ACECR, Tehran, Iran; ^3^Department of Biological Sciences, School of Science, Tokyo Metropolitan University, Tokyo, Japan; ^4^Graduate School of Science, Tokyo Metropolitan University, Tokyo, Japan

**Keywords:** Neurodegenerative Diseases, Tauopathy, Autophagy, Microtubule Binding Protein, Tau, Phosphorylation, Vesicle Trafficking

## Abstract

Abnormal deposition of misfolded proteins is a neuropathological characteristic shared by many neurodegenerative disorders including Alzheimer’s disease (AD). Generation of excessive amounts of aggregated proteins and impairment of degradation systems for misfolded proteins such as autophagy can lead to accumulation of proteins in diseased neurons. Molecules that contribute to both these effects are emerging as critical players in disease pathogenesis. Furthermore, impairment of autophagy under disease conditions can be both a cause and a consequence of abnormal protein accumulation. Specifically, disease-causing proteins can impair autophagy, which further enhances the accumulation of abnormal proteins. In this short review, we focus on the relationship between the microtubule-associated protein tau and autophagy to highlight a feed-forward mechanism in disease pathogenesis.

## Tau phosphorylation in physiology and disease

Misfolded tau protein is deposited in a group of neurodegenerative diseases called tauopathies, which include common forms of dementia such as AD and frontotemporal dementia [1,2]. Tau is a microtubule-binding protein whose primary physiological function is to regulate the assembly and stability of microtubules in neuronal axons [3]. However, tau detaches from microtubules and misfolds to form insoluble filaments in neurofibrillary tangles in the brains of patients with tauopathies [4-9]. Mutations of the MAPT gene, which encodes tau, are associated with dominant, inherited forms of frontotemporal dementia, indicating that tau abnormality contributes to disease pathogenesis [10]. Cellular and animal models of tauopathies suggest that elevated levels of tau protein are sufficient to cause neurodegeneration [11]. Thus, abnormal accumulation of tau is believed to cause neuron loss in diseased brains, and modulation of this accumulation has been suggested as a strategy to delay or prevent disease onset and progression. Tau contains a number of phosphorylation sites, and phosphorylation regulates both its physiological functions and pathological changes [12]. Phosphorylation of tau regulates its ability to interact with microtubules, its intracellular distribution, and its association with membranes [13]. Tau is natively unfolded, and phosphorylation alters its conformational status. Phosphorylation also affects its cleavage and further post-translational modifications [9]. In the brains affected by thauopathy, tau is highly phosphorylated at certain sites and its phosphorylation status is associated with the severity of pathology [2,6-8]. Conformational changes, mislocalization, and changes in protein interactions caused by pathological phosphorylation of tau have been suggested to slow down its degradation. A number of kinases, including proline-directed Ser/Thr kinases (SP/TP kinases) such as c-Jun N-terminal kinases (JNKs), cyclin-dependent kinase 5 (Cdk5), glycogen synthase kinase (GSK)-3β, and mitogen-activated protein kinase (MAPK), as well as non-SP/TP kinases including adenosine monophosphate-activated protein kinase (AMPK), calcium/calmodulin-dependent protein kinase II (CaMKII), checkpoint kinase 2, microtubule affinity-regulating kinase (MARK)/Par-1, NUAK family SNF1-like kinase 1 (NUAK1), p70S6K1, protein kinase A, and protein kinase C (PKC) phosphorylate tau [14-24]. Disruption of intracellular signaling involving these kinases may trigger hyperphosphorylation of tau in disease pathogenesis [25]. As described in the following section, some of these kinases also regulate autophagy.

###  Mechanism and Regulation of Autophagy 

Autophagy, or “self-eating”, is a preserved intracellular pathway via which accumulated or long-lived proteins and dysfunctional organelles undergo lysosomal degradation. Autophagy plays an essential role not only in cellular homeostasis and metabolism, but also in the physiopathology of several neurodegenerative disorders [26]. Autophagy can be categorized into three classes based on the mechanism by which cytoplasmic contents are targeted to the lysosome for degradation: microautophagy, macroautophagy and chaperone-mediated autophagy [27]. Macroautophagy enables the bulk degradation of cytosolic contents transferred to the lysosome by autophagosomes, whereas microautophagy is a process which results in the direct engulfment of cytoplasmic contents through lysosomal invagination. Chaperone-mediated autophagy degrades the cytosolic proteins that have the pentapeptide motifs. These proteins bind to heat shock cognate protein 70 and form complexes that are recognizable to a lysosomal chaperone-mediate autophagy receptor, LAMP2A. Consequently, the proteins are unfolded and translocated to the lysosome through lysosomal lumen [28]. In this article, we focus on macroautophagy, which is the major and well-known type of autophagy. Hereafter, we refer to macroautophagy simply as “autophagy”. The process of autophagy begins with formation of the phagophore, a cup-shaped double-membrane structure that surrounds autophagic substrates destined for degradation. Both edges of the phagophore elongate and close to form an isolated vacuole named an autophagosome. Thereafter, autophagosomes are transported toward the perinuclear region via dynein microtubule motors to fuse with lysosomes and to generate autolysosomes, which ultimately leads to degradation of their contents by lysosomal enzymes [29,30]. Autophagy is a conserved catabolic process that degrades cytoplasmic components in response to a lack of amino acids. Consequently, it is regulated by several signaling pathways that mediate nutrition sensing and stress responses. Mechanistic target of rapamycin (mTOR) plays critical roles in autophagic regulation in two different complexes mTORC1 and mTORC2. The primary function of mTORC2 is to modulate cellular morphology and cell migration, while mTORC1 plays a central role in the maintenance of energy homeostasis. mTORC1 suppresses autophagy and is regulated by upstream regulatory proteins that reflect the cellular levels of nutrients, growth factors, energy, and oxygen [31]. Inhibition of mTORC1 due to cellular stress, nutrient depletion, or low levels of energy or oxygen induces autophagy to promote cell survival by maintaining cellular homeostasis. Overactivation of mTORC1 signaling has been detected in AD brains and an animal model of AD [32,33]. AMPK signaling regulates autophagy in response to energy depletion [34]. Even a slight decrease in the cellular ATP/AMP ratio activates AMPK. Activation of AMPK can directly induce autophagy by inhibiting mTOR signaling and indirectly by stimulating ULK1 phosphorylation [34]. The inositol signaling pathway regulates autophagy independently of mTOR [35]. In this pathway, a reduction in the intracellular 1,4,5-inositol trisphosphate level induces autophagy.

###  Autophagy-Mediated Degradation of Tau

Tau degradation can be mediated by both the ubiquitin-proteasome pathway and autophagy pathway depending on its post-translational modifications, such as its phosphorylation state, folding, and solubility. Molecular chaperones recognize specific tau species and target them for proteasome-mediated degradation [36,37]. Induction of chaperones results in the selective clearance of tau phosphorylated at proline-directed sites such as pS202/T205 and pS396/S404 as well as conformationally altered tau [37]. Interestingly, tau phosphorylated at non-SP/TP sites (pS262/S356) evades this mechanism and remains stable [37]. On the other hand, a wide range of tau species can be degraded by autophagy. Induction of autophagy reduces the levels of tau phosphorylated at S262/356 that are not directed to proteasome [38,36]. Dolan *et al*. demonstrated that tau truncated at D421 is predominantly degraded by autophagy, while full-length tau is more prone to proteasomal degradation [39]. Another study suggested that induction of autophagy by trehalose in a mouse model of tauopathy decreases the amounts of insoluble tau and tau phosphorylated at T212/S214 (AT100) [40]. Altogether, long-lived or aggregation-prone tau species, such as phosphorylated tau, are more likely to be degraded by the autophagy pathway, while soluble monomeric and non-phosphorylated tau are degraded by the ubiquitin-proteasome pathway [41,42]. Thus, inhibition of autophagy may impair the degradation of high molecular weight tau species that accumulate in AD brains [43,44].

###  Mechanisms that Regulate Both the Generation of Neurotoxic Tau and Autophagy

Several kinases involved in autophagic regulation are also tau kinases or their regulators. GSK-3β phosphorylates tau at multiple sites and plays critical roles in tau toxicity [45]. Upregulation of the mTOR pathway not only downregulates autophagy but also elevates phosphorylation of tau via GSK-3β [46]. S6K downstream of mTOR also phosphorylates tau or affects degradation of tau; however, its roles in accumulation of tau remain controversial [32,38]. Lithium is a well-known GSK-3β inhibitor and stimulates autophagy through the inositol signaling pathway [47]. AMPK and members of the AMPK-related family of kinases, such as MARK/Par-1 and NUAK1, phosphorylate tau at S262 and S356 in the microtubule-binding repeats and induce its accumulation [48,24]. MARK4 inhibits mTORC1 activity and thus upregulates autophagy [49]. A sustained increase in the intracellular Ca^2+^ level activates Ca^2+^-sensitive tau kinases such as CaMKII and PKC [50]. It also induces activation of calpains, which cleave and thus activate the tau kinases GSK-3 and Cdk5 [51]. In addition, calpain cleaves the N-terminus of tau to generate neurotoxic fragments [52]. Although elevation of the intracellular Ca^2+^ level can induce or inhibit autophagy via several pathways [53], disruption of Ca^2+^ homeostasis has been implicated in disease pathogenesis and may affect these pathways to promote accumulation of tau.

###  Autophagy Impairment Caused by Pathological Tau Species

**Figure 1:**
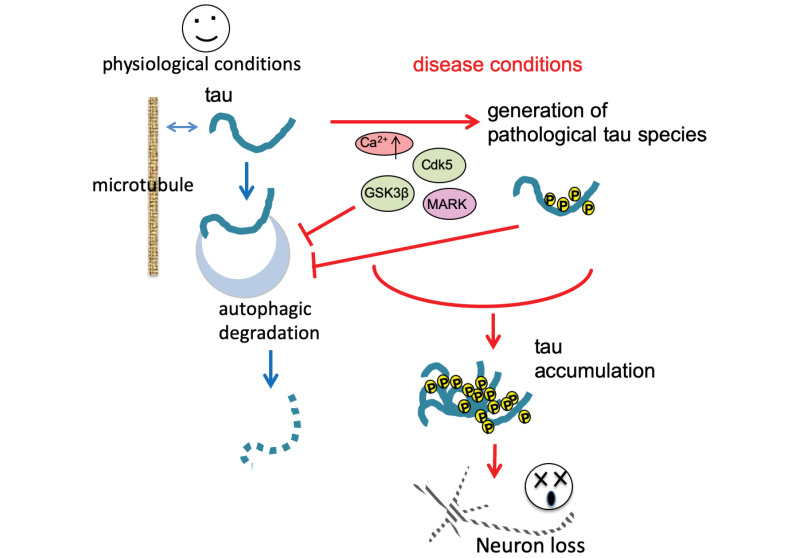
A possible feed-forward cycle between pathological tau species and inhibition of autophagy.

Accumulation of abnormal tau species may occur upstream of autophagic defects under disease conditions. Autophagy-mediated degradation requires stepwise maturation of autophagy vacuoles, which requires microtubule-dependent transport [54]. Tau regulates microtubule stability, while abnormal tau species can disrupt it. It has been reported that overexpression of tau impedes vesicle and organelle trafficking by disrupting the interactions between microtubules and motor proteins [55]. Expression of human wild-type or mutant tau causes deficits in axonal transport in transgenic mice [56,57] and *Drosophila *[58], as well as presynaptic defects in * Caenorhabditis elegans* [59]. These defects in membrane trafficking in neurons may impair the trafficking and maturation of autophagic vesicles. Neuronal autophagy is highly compartmentalized [60], and deficits in axonal transport caused by tau may significantly impact functionally distinct compartments such as synapses. Synaptic activity regulates autophagy in neurons, especially at synaptic terminals [54]. Synaptic dysfunction is one of the earliest pathological manifestations in AD and other tauopathies, and tau induces early synaptic deficits that precede synapse and neuron loss [61]. Abnormal tau species are missorted to pre- and postsynaptic terminals under disease conditions. It has been reported that tau in the presynaptic terminal reduces vesicle mobility and release rates via structural changes, Ca^2+^ dysregulation [62], or direct association with synaptic vesicles [63,64]. Tau disrupts the trafficking of postsynaptic receptors and thus suppresses postsynaptic neuronal activity [65]. Synaptic activity increases tau accumulation in lysosomes, and induction of synaptic activity stimulates the autophagic degradation of pathological tau levels, in mouse models of tauopathy [66]. These studies suggest that accumulation of abnormal tau caused by impaired autophagy can, in turn, suppress autophagic activity directly or indirectly (Figure-1).

## Conclusion

Accumulating evidence highlights the disruption of autophagy as a common theme in age-related neurodegenerative diseases with proteinopathy including AD [67]. This review focused on tau protein; however, other proteins deposited in diseased brains, such as α-synuclein and TDP-43, are also reported to interact with the autophagy pathway [68-70].

Enhancement of autophagy holds promise as a mechanism-based therapy to delay the onset and slow down the progression of diseases caused by abnormal protein accumulation [67]. However, autophagy has unique functions and regulatory mechanisms in neurons. Fine-tuning of autophagy is essential for normal neuronal development and functions such as synaptic transmission and memory formation [54]. Overactivation of autophagy can disrupt these processes and can also contribute to cell death under some disease conditions [71-75]. Further understanding of the regulatory mechanisms of neuronal autophagy and their disruption under pathological conditions will lead to a therapeutic application in the future.

## Acknowledgement

 This work was supported by a research award from the Japan Foundation for Aging and Health (to K.A) and a Grant-in-Aid for Scientific Research on Challenging Research (Exploratory) [JSPS KAKENHI Grant number 19K21593] (to K.A.) and. Authors thank Dr. Taro Saito for critical reading of this manuscript. Authors thank Dr. Mojtaba Farjam for the invitation to contribute this article.

## Conflict of Interest

 Authors declare that there is no conflict of interest regarding the publication of this article.
